# LEVERAGING TIME USE DATA TO EXPLORE MIGRANT HEALTH, SOCIALIZING, AND TECHNOLOGY USE

**DOI:** 10.1093/geroni/igae098.2126

**Published:** 2024-12-31

**Authors:** Amber DeJohn, Michael Widener, Bochu Liu, Xinlin Ma, Zhilin Liu

**Affiliations:** Florida State University, Tallahassee, Florida, United States; University of Toronto, Toronto, Ontario, Canada; Tongji University, Shanghai, Shanghai, China (People’s Republic); University of North Carolina, Chapel Hill, North Carolina, United States; Tsinghua University, Beijing, Beijing, China (People’s Republic)

## Abstract

Migrant populations tend to locate near other migrants upon arrival in their receiving country. These residential locations are the result of culturally specific social networks, and they result in differing social networks and cultural contexts relative to the native-born population and other migrant groups. Beyond their physical social networks, migrants also use information and communication technology (ICT) to maintain social connections in their sending country. During the COVID-19 pandemic, public health initiatives to discourage socializing (e.g., closing third places) generated concern about social isolation among older adults, especially due to its association with health. In Toronto, Canada, COVID-era lockdown initiatives were particularly prolonged. During this extended lockdown, we fielded a survey and time-use diary on a convenience sample of older Chinese migrants (n = 77) in the Greater Toronto Area. Using the single-day activity diaries, we grouped respondents using a k-means clustering approach, which resulted in four categories of socializing characteristics. We then used ANOVA tests and multinomial logistic regression to understand the geographic contexts for these socializing behaviors. Findings reveal that this older migrant community was largely socializing online, but a small group reported socializing in person. Regression results reveal that living conditions, physical health, and the location of social networks are associated with how migrants socialize. This study presents a novel method for exploring geographies and activities within the home in tandem.

